# Lateralized Brainstem and Cervical Spinal Cord Responses to Aversive Sounds: A Spinal fMRI Study

**DOI:** 10.3390/brainsci8090165

**Published:** 2018-08-31

**Authors:** Stephen D. Smith, Tiffany A. Kolesar, Jennifer Kornelsen

**Affiliations:** 1 Department of Psychology, University of Winnipeg, Winnipeg, MB R3B 2E9, Canada; s.smith@uwinnipeg.ca; 2 Department of Physiology and Pathophysiology, University of Manitoba, Winnipeg, MB R3E 0J9, Canada; Tiffany.Kolesar@umanitoba.ca; 3 Department of Radiology, University of Manitoba, Winnipeg, MB R3A 1R9, Canada

**Keywords:** spinal fMRI, emotion, spinal cord, auditory perception, International Affective Digitized Sounds (IADS)

## Abstract

Previous research has delineated the networks of brain structures involved in the perception of emotional auditory stimuli. These include the amygdala, insula, and auditory cortices, as well as frontal-lobe, basal ganglia, and cerebellar structures involved in the planning and execution of motoric behaviors. The aim of the current research was to examine whether emotional sounds also influence activity in the brainstem and cervical spinal cord. Seventeen undergraduate participants completed a spinal functional magnetic resonance imaging (fMRI) study consisting of two fMRI runs. One run consisted of three one-minute blocks of aversive sounds taken from the International Affective Digitized Sounds (IADS) stimulus set; these blocks were interleaved by 40-s rest periods. The other block consisted of emotionally neutral stimuli also drawn from the IADS. The results indicated a stark pattern of lateralization. Aversive sounds elicited greater activity than neutral sounds in the right midbrain and brainstem, and in right dorsal and ventral regions of the cervical spinal cord. Neutral stimuli, on the other hand, elicited less neural activity than aversive sounds overall; these responses were left lateralized and were found in the medial midbrain and the dorsal sensory regions of the cervical spinal cord. Together, these results demonstrate that aversive auditory stimuli elicit increased sensorimotor responses in brainstem and cervical spinal cord structures.

## 1. Introduction

The cervical spinal cord lies at an important nexus between the brain, which programs emotional motoric responses, and the peripheral nervous system that executes these responses. This region of the central nervous system consists of eight segments (C1–C8) that innervate the scalp, neck, shoulders, and upper limbs [1]. Ventral regions of the cord send projections that trigger responses in muscles and internal organs, whereas dorsal regions of the cord receive sensory feedback from these structures [1]. Although often thought of as a purely sensorimotor structure, previous research has demonstrated that viewing emotional stimuli leads to enhanced activity in both ventral and dorsal regions of the cervical spinal cord [2]. These responses are likely goal-directed, as emotional images depicting or implying responses by the upper limbs elicit more activity than emotional images related to lower-limb responses (which would require activity in the lumbar spinal cord [3]). In the current research, we examine whether emotion-dependent spinal cord activity can be elicited by salient stimuli that are auditory, rather than visual, in nature.

Earlier neuroimaging studies have delineated the brain areas involved in the perception of emotional sounds (see [4,5] for reviews). Several studies have highlighted the role of the amygdala in the processing of emotional sounds (e.g., [6,7,8,9]), a finding consistent with this structure’s role in rapid responses to emotionally salient stimuli [10,11]. The primary and secondary auditory cortices, as well as the superior temporal cortex, also reliably demonstrate enhanced responses to emotional stimuli (e.g., [12,13]). Recent data indicate that these temporal lobe structures interact during the perception of affective sounds. Using dynamic causal modeling of functional magnetic resonance imaging (fMRI) data, Kumar and colleagues [12] found that emotion-dependent activity in the auditory cortex triggers amygdalar responses to aversive sounds. This relationship appears to be reciprocal, with the amygdala also feeding back to the auditory cortex [14]. Importantly, the amygdala can also influence responses in the spinal cord. This modulation can occur via direct amygdalo-spinal projections [15] or through brainstem nuclei such as the parabrachial nucleus or the dorsal motor nucleus of the vagus [16]. Taken together, the enhanced activity in the amygdala and auditory cortices suggests that more neural resources are allocated to the processing of emotional sounds, a pattern similar to that found for the visual perception of emotional images [17,18]; based on neuroanatomical studies, it is possible that this preferential processing could modulate spinal cord activity.

Emotional sounds also activate neural regions associated with interoception, the ability to detect changes in internal somatosensory states [19,20]. Indeed, the insula and anterior cingulate cortex—which, together, comprise the brain’s salience network [21]—frequently respond to emotional sounds [9,22]. This pattern is noteworthy because numerous behavioural studies have demonstrated that aversive sounds elicit changes in electrodermal responses, heart rate, and pupil dilation [23,24,25]. Viscerosensory feedback associated with these psychophysiological changes inputs to dorsal nuclei in the spinal cord which, in turn, projects to the brain via the dorsal spinothalamic tract [26]. These pathways input to the nucleus of the solitary tract—a brainstem site of sensory, autonomic, and behavioural integration—along with the parabrachial nucleus and periaqueductal grey [27]. These brainstem regions extend to numerous cortical sites including the insula, amygdala, rostral and anterior midcingulate cortex, and the medial orbitofrontal cortex [28]. These neuroanatomical studies are supported by neuroimaging research demonstrated that autonomic responses in the body are associated with increased activity in the right dorsal anterior cingulate cortex and insula [29,30]. Based on these complementary lines of research, it is reasonable to conclude that the insula and anterior cingulate activity observed during the perception of emotional sounds is related to the body’s somatic responses to those stimuli [31].

Importantly, emotional auditory stimuli have also been shown to modulate activity in motoric regions of the brain. In a meta-analysis of neuroimaging studies, Früholz and colleagues [32] noted that the inferior frontal gyrus, basal ganglia, and cerebellum consistently show larger responses to aversive than neutral stimuli. This activity is likely related to response preparation (e.g., a withdrawal or freezing response), particularly when the perceived stimuli are simple nonhuman sounds rather than more complex vocal prosody [9,32]. This hypothesis is consistent with transcranial magnetic stimulation (TMS) studies showing that emotional stimuli increase the sensitivity of the corticospinal tract [33,34]. In contrast, suppressing sensorimotor activity via inhibitory TMS pulse sequences interferes with the processing of emotional vocalizations [35,36,37]. Together, these studies, using a variety of neuroimaging techniques, demonstrate that the processing of aversive emotional sounds involves motoric regions of the brain.

Given the sensorimotor nature of the brain’s responses to emotion-evoking sounds, we hypothesized that emotionally aversive auditory stimuli would also elicit greater levels of activity in the cervical spinal cord than would emotionally neutral sounds. This pattern of activity would indicate that the ventral spinal cord is initiating changes in upper-body musculature in response to the aversive sounds and that the dorsal spinal cord is receiving sensory information about these emotional responses.

## 2. Method

### 2.1. Participants

Nineteen undergraduate students were included in this study; two were excluded due to technical difficulties leaving 17 participants in the final analysis (13 female, 15 right-handed, age range 18–25). Ethics approval was obtained from the University of Manitoba’s Bannatyne Human Research Ethics Board (project identification code HS18235 (B2014:077); approval received 8 July 2014) and the University of Winnipeg’s Human Research Ethics Board (multi-site approval received 26 August 2014). All participants gave written informed consent and were screened for magnetic resonance imaging (MRI) safety prior to scanning. Participants receive a remuneration of $25.

### 2.2. Stimulus Materials

Stimuli consisted of 10 neutral and 10 negative emotion-evoking sounds, obtained from the International Affective Digital Sounds database (IADS, [38]). The Negative sounds consisted of the following IADS items: 115 (bees buzzing), 275 (male horror-movie-style scream), 276 (female horror-movie-style scream), 420 (car horns in a traffic jam), 422 (car accident), 424 (cars colliding), 600 (car crash with screaming), 709 (alarm clock ringing), 711 (sirens), and 719 (dentist drill). The Neutral sounds consisted of the following IADS items: 104 (dog panting), 245 (hiccup), 358 (eraser), 373 (scraping and rinsing dishes), 374 (rinsing dishes), 376 (lawnmower), 382 (shoveling snow), 698 (fireplace), 720 (brushing teeth), and 722 (non-threatening footsteps). The selection of the aversive stimuli was based on the norms accompanying the IADS database [38]. Neutral items were rated as being low in arousal and negative valence; specific neutral items were selected based on their semantic similarity to the emotional items (e.g., a human scream was matched with a human hiccup). *t*-tests comparing the average frequency and peak frequency of the emotional and neutral stimuli found no significant difference between the two groups (both *t*-values < 1). Stimuli were presented at 90 dB through the scanner room speakers. Although participants wore ear plugs for hearing protection in the noisy MRI environment, participants reported easily hearing the stimuli. Additionally, during a post-scan interview, participants were asked to list stimuli that they found particularly salient; all participants mentioned several sounds, suggesting that the stimuli were accurately perceived.

### 2.3. Experimental Design

Stimuli were presented in a boxcar design. In total, two spinal fMRI runs were collected: one run of neutral sounds and one run of negative emotion-evoking sounds. Each run consisted of three 60 s blocks of stimuli, interleaved by 40 s of rest. Ten sounds, each lasting for six seconds, were used per stimulus block. The order of the stimuli was randomized in each block for each participant.

### 2.4. fMRI Scanning Parameters and Preprocessing

MRI scanning was conducted using a 3-Tesla Siemens MAGNETOM Trio system (Erlangen, Germany) at the Winnipeg Regional Health Authority MRI satellite clinic. T_2_-weighted anatomical/functional cervical spinal cord images were acquired in sagittal orientation, extending from the corpus callosum to approximately T1 (single-shot fast spin echo (HASTE) sequence, TE/TR: 76/6750 ms per volume, FOV 280 × 210 mm, 1.46 mm × 1.46 mm resolution, 9 slices, 0 gap between slices, slice thickness: 2 mm, 50 volumes) (see [39] for a discussion of the challenges and advances in spinal cord functional imaging).

Spinal cord fMRI data were preprocessed with the custom-written Matlab scripts ‘spinalfMRI8’ (version 8, P.W. Stroman, Kingston, ON, Canada) [40,41] used in previous studies [2,3,42,43,44]. Slice timing correction and coalignment to correct for bulk motion using a non-linear 3D adjustment was applied. An automated normalization was performed that matched regions of the spinalfMRI8 spatially normalized template to the fMRI images by matching the corpus callosum, midbrain and pons in order to identify the cord in consecutive segments moving from rostral to caudal. Spatial smoothing with a 3D Gaussian filter was applied in the rostro-caudal direction at 2 mm.

### 2.5. fMRI Data Analysis

Spinal cord fMRI data were analyzed with the custom-written Matlab script ‘spinalfMRI8’ [40,41] used in previous studies [2,3,42,43,44]. Individual level data were analyzed using the General Linear Model (GLM) for each run to compare the stimuli blocks to the rest periods (*p* = 0.001). A second-level random effects analysis was performed to identify differences between the two conditions. Mean voxel-wise β-values from the individual-level GLMs were averaged and divided by the standard error of the mean to calculate *t*-statistics between conditions using a paired contrast (*p* = 0.01, contrast coefficients of −1, 1 for the Neutral and Negative conditions). A spatial extent method—where contiguous active voxels are considered more likely to be true positives and randomly distributed single voxels are considered more likely to be false positives—was applied to balance Type-I and Type-II errors. Thus, large clusters are likely to represent true activity while small, non-contiguous clusters are more likely to be false positive; for this reason, clusters consisting of five or fewer voxels have been excluded from results tables. Voxels exceeding threshold were displayed on the spatially normalized template, regions of activity were identified via comparison to the labelled normalized template, and significant voxels were manually counted. The center of gravity coordinates were extracted and represent the distance, in mm, from the pontomedullary junction (X = left to right; Y = ventral to dorsal; Z = rostral to caudal). 

## 3. Results

**Figure 1:**
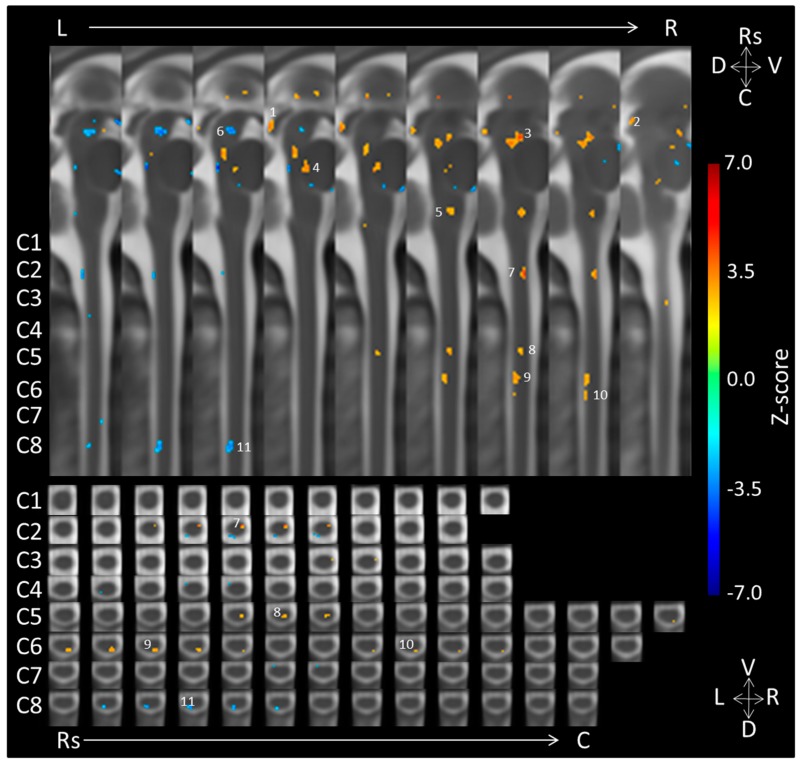
Group-level midbrain, brainstem and cervical spinal cord activity for the negative > neutral sounds contrast (*p* = 0.01; *n* = 17). Cervical spinal cord segments are labeled on the left of the figure for both sagittal and axial views. Voxels in orange represent significant activity for the negative as compared to the neutral condition and voxels in blue represent significant activity for the neutral as compared to the negative condition. Sagittal (above) and axial (below) views allow for Rs = rostral, C = caudal, D = dorsal, V = ventral, R = right, and L = left localization. Numbers 1–11 correspond to the clusters listed in Table 1 and Table 2.

The results from the contrasts of the negative and neutral fMRI runs can be found in Table 1 and Table 2 and in Figure 1. At the level of the midbrain, significant emotion-dependent activity was observed in regions of the right periaqueductal gray (PAG), right caudal inferior colliculus, and a region including the right rostral inferior colliculus and caudal superior colliculus. At the levels of the brainstem, enhanced responses were observed in the medial region of the pons, and the right medulla (in the region of the nucleus reticularis gigantocellularis). In contrast, neutral sounds led to greater activity in the left medial midbrain. When summed across the midbrain and brainstem regions, aversive sounds elicited activity in 156 voxels and neutral sounds elicited activity in 39 voxels.

The spinal cord results showed right-lateralized responses to negative emotional stimuli. The aversive sounds elicited more activity than neutral sounds in the ventral (motoric) regions of spinal cord segments C2 as well as in the dorsal (sensory) regions of C5 and C6. In contrast, neutral sounds led to greater activity in medial/left dorsal C8. When summed across the cervical spinal cord segments, aversive sounds elicited activity in 57 voxels in the right hemicord and neutral sounds elicited activity in 26 voxels in the medial/left hemicord.

## 4. Discussion

In the current study, aversive sounds led to a pronounced lateralization of activity in the midbrain, brainstem, and cervical spinal cord. These emotionally negative stimuli elicited right-lateralized activity in the medial and dorsal midbrain and brainstem and in both dorsal (sensory) and ventral (motoric) regions of several cervical spinal cord segments. Neutral stimuli, on the other hand, led to medial/left-lateralized activity in medial and dorsal (sensory) regions of the midbrain and spinal cord. Together, these data suggest that emotional sounds trigger activity in areas of the central nervous system that prepare an individual to perform an overt behavioural response.

It is important to note that the active voxels in our study reflect the results of a contrast analysis (i.e., Aversive vs. Neutral fMRI runs). Although the results of this analysis did not find any activity in the left brainstem or cervical spinal cord in response to aversive sounds, this does not mean that these structures were not active *at all.* Rather, it demonstrates that the amount of activity did not differ between the two conditions. This point is important; brainstem and ventral spinal cord activity isolated to one side of the body would not produce an evolutionarily useful motoric response to aversive stimuli. 

The cervical spinal cord data are consistent with earlier studies reporting that negative images elicit greater activity than neutral images in both dorsal and ventral regions of the cervical spinal cord [2,3,44]. The fact that aversive *auditory* stimuli also led to increased spinal cord activity suggests that the emo-motoric responses found in earlier research are part of a general threat-response system rather than being modality-specific. Future research using additional sensory modalities is necessary to determine whether any aversive sensory experience is sufficient to stimulate spinal cord neurons or whether this system reacts only to stimuli that are threatening in nature. Additional studies could also clarify whether these sensorimotor emotional responses are triggered by emotional valence (i.e., positive vs. negative) or arousal, or to an interaction of these two stimulus characteristics.

The observed activity in the midbrain and brainstem is also likely a sensorimotor response. The enhanced activity in the inferior colliculus is almost certainly related to this structure’s role in orienting to auditory stimuli [45,46]. Emotional stimuli are more salient than neutral stimuli and should, therefore, elicit more activity in this region than neutral stimuli. Projections from the inferior colliculi could then influence activity in other subcortical brain regions including the pulvinar nucleus and the amygdala [32], thus allowing the individual to allocate more attention toward these aversive stimuli.

The activity in the PAG is consistent with this structure’s role in integrating affective, autonomic, and motoric information [47]. Animal studies have noted that the lateral/dorsolateral column of the PAG is related to fight-or-flight responses [48]. These responses are mediated by descending projections to more caudal brainstem structures including the paragigantocellularis lateralis nucleus of the rostral ventrolateral medulla [49], a structure that appeared to show emotion-dependent activity in the current study. Given that previous neuroimaging studies have detected PAG activity in response to threatening stimuli [50] and frustration [51]—both of which involve changes in muscular responses (e.g., [52])—it seems reasonable to conclude that the PAG responses in the current study are also emo-motoric in nature.

The aversive auditory stimuli also elicited activity in the dorsomedial region of the pons. Although the current scanning parameters lacked the precision to isolate specific pontine nuclei, the dorsomedial subregion of the pons does include the pedunculopontine tegmental nucleus, a cholinergic structure that modulates both arousal and motoric responsivity in combination with noradrenergic outputs from the locus coeruleus [53,54]. These ascending projections have been shown to modulate cortical responses in the supplementary motor area and motor cortices [53]. They also influence arousal by disinhibiting thalamic nuclei that project to the cortex [55]. It is possible that such activity was elicited by the aversive stimuli used in the current study.

An obvious question that emerges from the current data relates to the specific nature of the motoric response. Put simply, what *type* of motoric response is being planned? Although the exact nature of this behavioral response is difficult to assess within the physical confines of an MRI scanner, previous behavioral studies suggest two likely possibilities. The first is that the participants wished to avoid or withdraw from the aversive stimuli. If this view is accurate, then the observed activity in the brainstem and cervical spinal cord would reflect a form of “action preparedness” that would allow the individual to make a rapid and efficient response to a threatening stimulus [56,57]. Alternatively, the changes in brainstem and spinal cord activity could be associated with a “freezing” response. Freezing involves tensing muscles throughout the body in order to reduce or eliminate any movement that could be detected by a predator [58]. Although the participants in the current study were not directly threatened, freezing behaviors have been identified in experimental paradigms using human participants [59]. Given that the MR scanning environment limited the number of overt behaviors that could be performed by participants, freezing may have been a potential motoric response to the aversive stimuli. Consistent with this view, freezing has been linked with increased activity in the PAG [60,61], a region that responded to aversive stimuli in the current study. It is also possible that both forms of motoric behavior occurred simultaneously, with freezing occurring while a behavioral response was being planned [62]. Future research utilizing psychophysiological and electromyographical (EMG) measurement techniques will be necessary to test these possibilities.

An intriguing element of the current research is that the neural responses to aversive stimuli were lateralized to the right side of the midbrain, brainstem, and spinal cord. At first glance, it is tempting to assume that this activity is part of an overall pattern of laterality. Indeed, numerous studies have highlighted the importance of right-hemisphere structures in the perception of emotionally negative and/or avoidance-related stimuli (see [63] for a review of competing theories of the lateralization of emotion). However, such a conclusion would be premature. The numerous ascending and descending pathways of the central nervous system decussate at different levels of the brainstem. It is unclear, therefore, whether the lateralized activity in the brainstem and spinal cord is an extension of hemispheric differences in the other brain areas such as the amygdala or prefrontal cortices (e.g., [8,29,64]). Future investigations using functional MRI of the brain and spinal cord—with parameters optimized to disambiguate the activity of different brainstem nuclei—would allow us to better characterize the nature of these right-lateralized responses. 

An additional possibility is that the right-lateralization of motoric responses were due to the fact that 88% of our participants were right-handed. Although there is little evidence that handedness greatly affects the lateralization of emotional perception in the brain [65], it is possible that the motoric programs resulting from this perception might be biased toward an individual’s dominant hand. This possibility could be tested in future studies by scanning equal numbers of left- and right-handed participants. 

Although the current study provides novel information about how the brainstem and cervical spinal cord process aversive sounds, there are limitations worthy of discussion. First, only aversive and neutral items were used. Although it would be interesting to investigate whether different types of emotions—particularly happiness—would trigger motoric responses, pilot testing of the IADS stimuli suggested that the aversive stimuli were perceived as being more salient and threatening. Therefore, for our initial study of spinal cord responses to emotions, we focused on stimuli that were likely to elicit a motoric response such as withdrawal or freezing. Second, the stimuli used in the current study consisted primarily of environmental sounds. A previous meta-analysis of neuroimaging studies noted that different types of emotional auditory stimuli recruit slightly different neural networks [32]. That said, this same analysis identified a core network of brain areas recruited across emotional stimulus categories; this network includes the amygdala and sensorimotor structures relevant to the current study. Therefore, we can be relatively confident that similar patterns of spinal cord activity would be detected if different categories of emotional sounds were used. 

## 5. Conclusions

In conclusion, the results of the current study extend our understanding of the neural networks involved in the perception of aversive sounds. Future studies examining the sensitivity of these midbrain, brainstem, and spinal cord regions to manipulations of emotional valence and arousal will help to further refine our understanding of how the central nervous system responds to emotional auditory stimuli.

## Figures and Tables

**Table 1:** Location of brainstem activity in the contrast of the negative sounds and neutral sounds.

Location	Side	Dorsal/Ventral	Coordinates (mm)			Voxels
			X	Y	Z	
***Negative > Neutral***						
1. Midbrain (caudal IC)	Right		−2	21	27	24
2. Midbrain (rostral IC/caudal SC)	Right		−7	18	30	8
3. Midbrain (Periaqueductal Gray)	Right	Medial	−3	9	20	81
4. Pons	Medial	Dorsal	−1	6	10	23
5. Medulla	Right	Medial	−4	5	−8	20
***Neutral > Negative***						
6. Midbrain	Medial/Left	Medial	1	7	26	39

IC = inferior colliculus; SC = superior colliculus. Results are presented at *p* < 0.01, with a cluster threshold of ≥5. Coordinates are position, in mm, relative to the pontomedullary junction for the center of mass. The cluster number corresponds to Figure 1.

**Table 2:** Location of spinal cord activity in the contrast of the negative sounds and neutral sounds.

Location	Side	Dorsal/Ventral	Coordinates (mm)			Voxels
			X	Y	Z	
***Negative > Neutral***						
7. C2	Right	Ventral	−4	4	−34	13
8. C5	Right	Dorsomedial/medial	−3	5	−67	13
9. C6	Right	Dorsal	−4	7	−78	26
10. C6	Right	Dorsal	−5	8	−85	5
***Neutral > Negative***						
11. C8	Medial/Left	Dorsal	1	8	−107	26

Results are presented at *p* < 0.01, with a cluster threshold of ≥5. Coordinates are position, in mm, relative to the pontomedullary junction for the center of mass. The cluster number corresponds to Figure 1.
